# Supplementary material for: Exfoliating effect of β-glycyrrhetinic acid on plaque inducing gingivitis: Comparison with cetylpyridinium chloride
Source: PLoS One. 2026 May 28;21(5):e0348495. doi: 10.1371/journal.pone.0348495 (PMC13218510; doi:10.1371/journal.pone.0348495)
Supplement: S1 Table — (DOCX) [file pone.0348495.s001.docx]

**S1 Table.** Test groups and test medium

| Reagent concentration in BHI medium | | | |
| --- | --- | --- | --- |
| Test group | BGA | CPC | DMSO (as control) |
| BGA | 0.0128% |  |  |
| CPC |  | 0.004% |  |
| DMSO | 5.000% | 5.000% | 5.000% |

BHI: Brain heart infusion medium, BGA: β-glycyrrhetinic acid, CPC: cetylpyridinium chloride, DMSO: dimethyl sulfoxide
