# Supplementary figures and images for: Exfoliating effect of β-glycyrrhetinic acid on plaque inducing gingivitis: Comparison with cetylpyridinium chloride
Source: PLoS One. 2026 May 28;21(5):e0348495. doi: 10.1371/journal.pone.0348495 (PMC13218510; doi:10.1371/journal.pone.0348495)

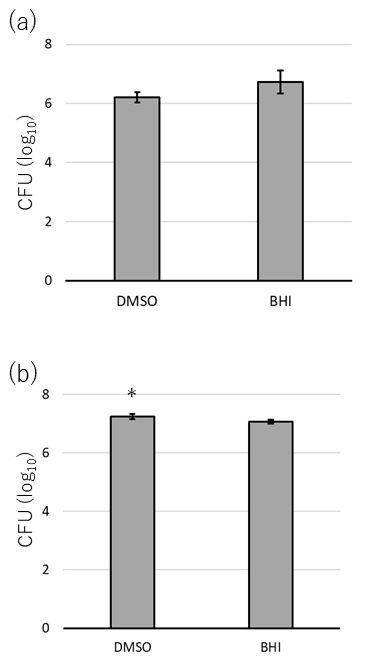

Supplement: S1 Fig — The formed biofilm was incubated for 6 h in Brain Heart Infusion medium (BHI) with and without 5.0% DMSO. CFU in (A) the biofilm remaining and (B) supernatant were measured. These experiments were carried out in triplicate. Error bars denote standard deviation. Comparisons with and without DMSO were performed using the t-test.* P < 0.05. (TIF) [file pone.0348495.s002.tif]

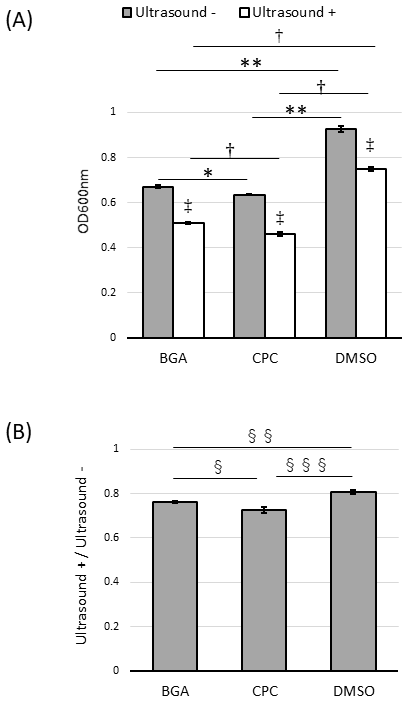

Supplement: S3 Fig — Biofilm biomass following incubation in Brain Heart Infusion medium containing minimum inhibitory concentration of BGA and CPC was determined by crystal violet assays before and after sonication. Absorbance at OD600 (A) and ratio of absorbance at OD600 before and after sonication (B). All the experiments were carried out in triplicate. Error bars denote standard deviation. *P < 0.01, ** P < 0.001 (Ultrasound -) †P < 0.001 (Ultrasound +) § P < 0.05, §§P < 0.01, §§§P < 0.001. Comparisons before and after ultrasound were performed using t-test. ‡ P < 0.001. (TIF) [file pone.0348495.s004.tif]
