# Supplementary material for: Exfoliating effect of β-glycyrrhetinic acid on plaque inducing gingivitis: Comparison with cetylpyridinium chloride
Source: PLoS One. 2026 May 28;21(5):e0348495. doi: 10.1371/journal.pone.0348495 (PMC13218510; doi:10.1371/journal.pone.0348495)
Supplement: Attachment — Submitted filename: Response to Reviewers.docx [file pone.0348495.s006.docx]

EMID: beb8b9fc8494d88d

Reply to the Editor

Thank you for your important comments and suggestions.

We have addressed them as follows.

1. Study Scope and Conceptual Framing

- The mechanistic interpretation and terminology require greater precision and consistency throughout the manuscript.

We revised the manuscript to clarify the differences between BGA and CPC (line 361-362 and 370-371).

2. Major Methodological Limitations

- The limitations of these three models ( Static vs. dynamic biofilm conditions; exposure duration; pooling of plaque samples) must be discussed.

This in vitro study to verify the effectiveness of BGA against dental plaque differs from clinical studies in several ways. One key difference was that biofilms derived from supragingival plaque on polystyrene plates were used in this study. This allowed testing with uniform biofilms while still replicating the complex bacterial flora found in the oral cavity. Consequently, BGA and CPC could be compared from various perspectives, including biofilm quantity, viable bacteria count, and bactericidal rate, under identical exposure conditions to the test substances. However, while enzymatic degradation of biofilm was observed on polystyrene plates, some reports indicate no significant difference from placebo formulations in vivo. Conversely, Yamashita et al. reported reduction in plaque adhesion after one week of using a toothpaste containing 0.1% BGA. The use of oral compositions containing BGA is expected to inhibit plaque adhesion. In contrast, this study exposed biofilms to a low concentration of 128 μg/ml for 6 hours. This was done to test at the MIC level of BGA, aiming to clarify the mechanism of its effect. We monitored the viable bacteria and biofilm amount over time, and the effect reached a plateau after 6 hours. BGA has also been reported to exhibit bacteriostatic activity and plaque formation inhibition. Therefore, the extent to which the exfoliation effect demonstrated in this study contributes remains unclear. Further verification, such as investigating plaque removal efficacy in vivo, is considered necessary.

This description has been added to the “DISCUSSION” (line 399-417).

Another reason for pooling of plaque sample was that it enabled consistent experimentation with a uniform biofilm, requiring minimal sample collection per individual.

This description has been added to “MATERIALS AND METHODS” (line 119-120).

3. Critical Scientific Concerns

a. Distinction between "killing" and "removal" is unclear.

We standardized terminology throughout the manuscript.

b. Mechanistic evidence is insufficient.

In the case of *Vibrio cholerae biofilms*, it has been reported that BGA exposure alters the composition of exopolysaccharides. This may have influenced biofilm exfoliation. However, the present study could not demonstrate chemical or structural changes in the biofilm following BGA exposure. Investigating these aspects would provide more definitive evidence regarding this exfoliation effect.

We added this limitation and the need for further study to the “DISCUSSION” (line 374-398).

4. Experimental Gaps and Required Controls

- To benchmark the exfoliating effect of BGA, include a positive control.

Although different from these mechanisms, enzymes are sometimes used to promote plaque removal. Comparing their effects is considered valuable for verifying clinical efficacy and should be pursued in future studies.

This statement was added to “DISCUSSION” (line 383-386).

- The manuscript must clearly confirm that DMSO concentrations used as vehicle controls do not independently affect biofilm integrity.

We confirmed that 5% DMSO did not reduce viable bacterial counts (S1 Fig. (A) and (B)).

This text has been added to the “RESULTS” (lines 238-239).

- To substantiate the claim of scaffold disruption, incorporation of EPS-targeted staining in CLSM analysis is recommended

We intend to make the direct visualization of matrix alteration for future research.

This description has been added to “DISCUSSION” (line 378-379).

5. Revision Guidance

- Reframe the Discussion to emphasize biofilm structural modification rather than general antimicrobial activity, unless bactericidal effects are conclusively demonstrated.

We revised the manuscript to clarify the differences between BGA and CPC (line 361-362, 368, 370-371, and 390-392).

- Address all reviewer comments in a detailed, point-by-point response document, and explicitly respond to the concerns that led to the recommendation for rejection.

This Response to Reviewers addresses all comments and describes how they were resolved.

Reply to Reviewer 1

Thank you for your important comments and suggestions. We have addressed them as follows.

1. Does the degree of turbidity reflect the biofilm exfoliation effect?

Before exposing BGA and CPC to biofilms, the supernatant was removed and washed, suggesting that suspended solids in the supernatant originated from biofilms. Furthermore, the concentrations of BGA and CPC were at MIC levels, indicating that bacterial growth in the supernatant was inhibited. Therefore, we interpret turbidity as reflecting biofilm detachment.

This text has been added to the “RESULTS” (line 317-322).

2. Please discuss the potential application of BGA in oral care products. Is the biofilm detachment the only clinically relevant factor?

Yamashita et al. reported that reduction in plaque adhesion after one week of using a toothpaste containing 0.1% BGA. BGA has been reported to exhibit bacteriostatic activity and plaque formation inhibition. Therefore, the extent to which the biofilm exfoliating effect demonstrated in this study contributes remains unclear. Further verification, such as investigating plaque removal efficacy in vivo, is considered necessary.

This text has been added to the “DISCUSSION” (lines 407-417).

3. Please discuss the rationale and limitations of the concentration settings and the interpretation of MIC-based comparisons.

Yamashita et al. reported that reduction in plaque adhesion after one week of using a toothpaste containing 0.1% BGA. In contrast, we exposed biofilms to BGA at 128 ug/mL for 6 hours to test BGA at its MIC level and to help clarify its mechanism.

This text has been added to the “DISCUSSION” (lines 407-412).

4. Clarify that the discussion of quorum sensing is speculative.

We added statements indicating that quorum sensing as a mechanism for the BGA detachment effect is a hypothesis and has not been confirmed (lines 382-383 and lines 390-391).

5. Add arrows or brief annotations indicating detached biofilm aggregates in Fig. 6.

We added arrows indicating detached biofilm aggregates in Fig. 6.

6. Add the detection limit of the CFU assay in Fig. 5B.

We added that the detection limit of the CFU assay is 47.6 to the “RESULTS” (line 242 and lines 306-307).

Reply to Reviewer 2

Thank you for your important comments and suggestions. We have addressed them as follows.

1. Revise all claims of "bactericidal action" throughout the manuscript.

We revised the manuscript to state that the primary effect of BGA is bacteriostatic (line 226, line 361, line 368, lines 370-371 and line 427).

2. Correct the misreporting of bacterial strains.

We have corrected the misreporting of bacterial strains (line 74, line 77, lines 99-100, line 218, and lines 227-229).

3. Resolve the contradiction involving the phrase "data not shown."

Because the MIC of CPC for supragingival plaque was 40 ug/mL, we deleted "data not shown" (line 233).

4. Tone down translational claims (lines 394-395).

Given that dental plaque accumulation contributes to periodontal disease, we think that BGA helps prevent gingivitis and periodontitis by reducing plaque levels. We revised the statement to be more cautious regarding clinical implications (lines 429-430).

5. Clarify whether assumptions for parametric tests were checked.

Normality was tested by Shapiro–Wilk test.

This was added to “MATERIALS AND METHODS” (line2 202-203).

6. Correct typos and grammatical errors.

We corrected typos and grammatical errors (line 226, line 307, line 361, line 368, lines 370-371 and line 427).

Reply to Reviewer 3

Thank you for your important comments and suggestions. We have addressed them as follows.

1. Title: Revise the classification of gingivitis.

In accordance with the American Academy of Periodontology and the European Federation of Periodontology classification, we replaced "marginal gingivitis" with "plaque-induced gingivitis" (line 1, line 4, line 44, line 45, and line 51). We also added Ref. 8 as supporting evidence (lines 455-457).

2. Introduction: Include that gingivitis progression can lead to periodontitis.

We added Ref. 8, which states that gingivitis is a precursor of periodontitis (line 44, and lines 455-457).

3. Introduction: Is there concern regarding the emergence of BGA-resistant oral bacteria?

Concerns about the emergence of CPC-resistant oral bacteria exist, and while no reports of BGA- resistant bacteria have emerged, the possibility cannot be ruled out. Few anti-biofilm agents target quorum sensing, and they are currently being researched as a new approach against drug-resistant bacteria. Therefore, we expect BGA to be a new anti-plaque agent.

This text has been added to the “DISCUSSION” (lines 392-396).

4. Materials and Methods: Why were samples not collected from patients with gingivitis?

To investigate the efficacy in preventing gingivitis and periodontitis, bacterial communities adjacent to the gingival margin were collected from participants who did not have gingivitis or periodontitis.

This text has been added to “MATERIALS AND METHODS” (lines 112-114).

5. Materials and Methods: How was the MBC of 2048 ug/mL measured in Table 1?

When the MBC was 1024 ug/mL or higher, the BGA concentration was adjusted to 0, 256, 512, 1024, 2048, and 4096 μg/ml, and the experiment was repeated using the same procedure.

This text has been added to Materials and Methods (lines 144-146).

6. Materials and Methods: Justify using OD_600_ of supernatants as the amount of detached biofilm.

Before exposing BGA and CPC to biofilms, the supernatant was removed and washed, suggesting that suspended solids in the supernatant originated from biofilms. Furthermore, the concentrations of BGA and CPC were at MIC levels, indicating that bacterial growth in the supernatant was inhibited. Therefore, the turbidity in the supernatant was likely due to detachments from the biofilms, rather than bacterial growth in the supernatant.

This text has been added to the “RESULTS” (lines 317-322).

7. Materials and Methods: How were the 24 h/6 h incubation periods determined?

Supragingival plaque was incubated for 24 hours to form biofilms, consistent with a previous study. Reference was added at line 151, line 159, line 175, line 236 and lines 569-571.

For BGA exposure, we monitored viable bacteria and biofilm amount over time, and the effect plateaued after 6 hours; therefore, we set the exposure time to 6 hours.

This text has been added to the “DISCUSSION” (lines 412-413).

8. Materials and Methods: "Prevotella denticola JP2" or "Aggregatibacter actinomycetemcomitans JP2"?

We revised this to Aggregatibacter actinomycetemcomitans JP2 (line 228).

9. Materials and Methods: "Prevotella denticola ATCC25611" or "Prevotella intermedia ATCC25611"?

We revised this to *Prevotella intermedia* ATCC25611 (line 229).

10. Results: Was it a comparison with the BGA group or the DMSO group (P15L242)?

This was a comparison with DMSO alone. We revised the sentence to: "which was significantly (97.8%) lower (P<0.001, Fig. 1) than DMSO alone." (lines 242-244).

11. Results: Revise the sentence "both CPC and BGA reduced biofilm formation to the same degree" (P18L294-295).

We revised the sentence to: "Both CPC and BGA reduced biofilm formation". (line 296).

12: Discussion: OD_600_ could be affected by bactericidal and bacteriostatic properties. How were these excluded?

Before exposing BGA and CPC to biofilms, the supernatant was removed and washed, suggesting that suspended solids in the supernatant originated from biofilms. Furthermore, the concentrations of BGA and CPC were at MIC levels, indicating that bacterial growth in the supernatant was inhibited. Therefore, the turbidity in the supernatant was likely due to detachments from the biofilms, rather than bacterial growth in the supernatant.

This text has been added to the RESULTS (lines 317-322).

13. Discussion: Discuss other limitations (s the specific concentrations used, fixed incubation times, or generalizability of results from polystyrene plates to complex oral surfaces).

This is in vitro study to verify the effectiveness of BGA against dental plaque, differs from clinical studies in several ways. One key difference was that biofilms derived from supragingival plaque on polystyrene plates were used in this study. This allowed testing with uniform biofilms while still replicating the complex bacterial flora found in the oral cavity. Consequently, BGA and CPC could be compared from various perspectives, —including biofilm quantity, viable bacteria count, and bactericidal rate, —under identical exposure conditions to the test substances. However, while enzymatic degradation of biofilm was observed on polystyrene plates, some reports indicate no significant difference from placebo formulations in vivo. Conversely, Yamashita et al. reported reduction in plaque adhesion after one week of using a toothpaste containing 0.1% BGA. The use of oral compositions containing BGA is expected to inhibit plaque adhesion. In contrast, this study exposed biofilms to a low concentration of 128 μg/ml for 6 hours. This was done to test at the MIC level of BGA, aiming to clarify the mechanism of its effect. We monitored the viable bacteria and biofilm amount over time, and the effect reached a plateau after 6 hours. BGA has also been reported to exhibit bacteriostatic activity and plaque formation inhibition. Therefore, the extent to which the exfoliation effect demonstrated in this study contributes remains unclear. Further verification, such as investigating plaque removal efficacy in vivo, is considered necessary.

This description has been added to “DISCCUTION” (line 399-417).

14. Comment: English editing is required throughout the manuscript.

We obtained professional English editing and re-checked the manuscript to correct remaining errors. We also corrected specific terms (e.g., “*Pseudomonas aeruginosa*” at line 74, “Klebsiella pneumoniae” at line 77, “*Aggregatibacter actinomycetemcomitans*” at lines 99-100, and “*Streptococcus salivarius* JCM5707” at line 218) and removed "Frankrin" and "data not shown," as appropriate.
